# Borrelia burgdorferi exposure in coyotes: an indicator of B. burgdorferi levels in urban versus rural environments

**DOI:** 10.18849/ve.v7i1.444

**Published:** 2022-02-16

**Authors:** Laura Shultz+, Erik Fausak+

**Keywords:** BORRELIA BURGDORFERI, BORRELIOSIS, CANIS LATRANS, COYOTES, LYME DISEASE, RURAL AREAS, SEROPREVALENCE, URBAN AREAS

## Abstract

PICO question

Do wild coyotes in the US that are in an urban habitat compared to a rural habitat have a higher prevalence of *Borrelia burgdorferi* seroconversion?

Clinical bottom line

Category of research question

Prevalence

The number and type of study designs reviewed

Two papers, both utilising a cross-sectional study design

Strength of evidence

Zero

Outcomes reported

The relevant studies provide very limited to no evidence towards answering this PICO question. In one, while the absolute percentage of *Borrelia*-antibody-positive canines (including dogs in addition to coyotes) is higher in metropolitan areas, the effect was not found to be statistically significant, possibly due to their small sample sizes. In the second study, prevalence of antibodies against *Borrelia* was compared between different rural habitats, but no urban coyotes were tested as a comparison and thus the PICO question cannot be evaluated

Conclusion

There is a knowledge gap concerning the prevalence of *Borrelia* in coyotes and how it differs between urban and rural environments. Wild coyotes could be used as a sentinel species of Lyme disease activity and to assess potential for domestic pet and human infections, which would inform clinical differential diagnoses as well as testing and vaccination recommendations. More studies are needed before this PICO question can be answered in a confident manner

## How to apply this evidence in practice

The application of evidence into practice should take into account multiple factors, not limited to: individual clinical expertise, patient’s circumstances and owners’ values, country, location or clinic where you work, the individual case in front of you, the availability of therapies and resources.

Knowledge Summaries are a resource to help reinforce or inform decision making. They do not override the responsibility or judgement of the practitioner to do what is best for the animal in their care.

## Clinical scenario

A dog presents with fever, painful/swollen joints, shifting lameness, lack of appetite, lethargy, and swollen lymph nodes. The patient is not up to date on parasite preventatives, and Lyme disease is suspected. Knowing the prevalence of *Borrelia burgdorferi* in the area and whether the patient lives in an urban or rural environment could influence how likely that diagnosis is and if it should be given serious consideration. Coyotes occupy urban, rural, and wilderness environments and can serve as a sentinel species to indicate *Borrelia *disease levels and geographical trends to help inform diagnoses and vaccination recommendations.

## The evidence

While cross-sectional studies provide a high level of evidence for prevalence questions assessing exposure to this pathogen, neither of the two studies evaluated here were directly investigating whether *Borrelia burgdorferi* levels vary between urban and rural environments in wild coyotes of the United States. Due to this, the evidence that they provide towards the PICO question of interest is extremely limited.

In the Olson et al*. *study from 2000, seroprevalence was determined for canines including both dogs and coyotes in San Diego County, CA, allowing an assessment of risk for human cases of Borreliosis to be conducted. No statistical association was found between disease prevalence and lifestyle (rural vs urban vs indoors) of the canine host, but prevalence of *B. burgdorferi *antibodies was higher in metropolitan dogs, with the lowest prevalence being found in the most natural habitat; they postulated this could be due to importation of seropositive animals by high mobility owners or ‘brush-border habitat’ styles in urban landscaping supporting tick populations (Olson et al*., *2000). However, when looking solely at coyotes, the sample size is small (n = 83), and there was only one positive individual, which was from a rural environment (Olson et al.*,* 2000).

Five years later, Foley et al*. *(2005) published a study that looked deeper at variation in *B. burgdorferi* seroprevalence by vegetation type and climate within *Ixodes pacificus­*­-infested counties of California. While Foley et al. (2005) did not directly classify the tested individuals as urban or rural, ‘urban’ was a habitat option and urban areas are included in mapped visualisations of the data; examining their maps, it appears no or few coyotes from urban habitats were tested. With no tested urban coyotes clearly identifiable, this study does not allow us to answer the target PICO question but does explore variation within rural environments at a finer scale (Foley et al.*, *2005). Prevalence was higher in areas with higher rainfall as well as in blue oak woodland, montane hardwood, and redwood vegetation habitats, with seasonal peaks in June – July and November – April (Foley et al.*, *2005).

There were no papers that directly addressed this PICO question, which identifies a gap in knowledge where further research is needed.

## Summary of the evidence

**Olson et al. (2000):** 

Population:	Canines: Dogs (*Canis familiaris*) and coyotes (*Canis latrans*).
Sample size:	1000 canines (917 dogs, 83 coyotes).
Intervention details:	Canine sera were obtained from local veterinarians, trappers, animal shelters, and humane societies between March 1992 and March 1993. Urban or Rural ‘Lifestyle’ designation was included in the data collected by the investigators. Sera were tested by ELISA for *Borrelia burgdorferi *antibodies with results being validated via Western blot test and/or indirect fluorescent antibody (IFA) tests at reference laboratories. Criterion for positive ELISA: spectrophotometrically three standard deviations above the mean optical density of samples from control animals. Criterion for positive Western blot test: presence of 5/10 most common antigen IgG bands. Criterion for positive IFA: >1:128 of the equivalent when correlating for interlaboratory variability.
Study design:	Cross-sectional study design.
Outcome studied:	The objective of this study was to determine the seroprevalence of *B. burgdorferi *in San Diego county as assessed in canines, who can serve as a sentinel for human risk of Lyme disease cases. Seroconversion was measured by ELISA and confirmed via Western blot test and/or IFA at a reference laboratory.
Main Findings (relevant to PICO question)	For wild coyotes, no association with lifestyle (urban vs rural) was found (P = 1.0 for coyotes). Only one coyote (rural) tested positive for Lyme disease. While no significance of *burgdorferi* occurred in the coyote population, the domestic dog data showed some interesting outcomes: The location considered the wildest, Camp Pendleton, a US Marine Corps training area, had the lowest seroprevalence in dogs (0.5%). Seroprevalence of dogs in the metropolitan sector was the highest (4.6%). The authors postulate this could be due to importation of seropositive animals by high mobility owners or ‘brush-border habitat’ styles in urban landscaping supporting tick populations.
Limitations:	When considering only coyotes, the sample size is small (total n = 83, seropositive n = 1), especially given that Borreliosis is rare. The low number of positives limited the power of subset analysis. *** *** Prevalence may be overinflated due to cross-reactivity of antibodies. The authors did not utilise a vaccine free population, though they state only three dogs in the study had received the vaccine.

**Foley et al. (2005):** 

Population:	Coyotes in *Ixodes pacificus *infested counties of California.
Sample size:	215 total coyotes.
Intervention details:	215 coyotes were trapped by USDA Wildlife Services as part of a predator control program and convenience samples were taken from these animals for analysis. 170 were tested for *Borrelia burgdorferi*, as blood from 45 coyotes from one county was not available when *burgdorferi* testing was performed. Coyotes were split into several subsets including county, habitat, and month for analyses with varying sample sizes from 1 to 57. ‘Urban’ was a habitat option and urban areas are included in mapped visualisations of the data; examining the maps, there are no urban coyotes easily identifiable. Blood was either collected ante-mortem into ethylenediamine tetraacetic acid (EDTA) or heart clot blood was collected post-mortem. Sera was tested via indirect fluorescent antibody (IFA) for *burgdorferi*, with positive-IFA sera verified with Western blot testing. Criterion for positive IFA: a positive cut off of 1:25 was used to indicate further testing by Western blot test. Criterion for positive Western blot test: presence of three or more diagnostic IgG bands.
Study design:	Cross-sectional study design.
Outcome studied:	The aim of this study was to examine spatial and temporal relationships at a smaller-scale to understand ecological drivers of disease among *Anaplasma phagocytophilum* and *B. burgdorferi*-exposed coyotes. Prevalence of disease between different vegetation type and climate in *Ixodes pacificus* infested counties of California was investigated. Seroconversion for *B. burgdorferi* was tested via IFA and confirmed via Western blot test.
Main Findings (relevant to PICO question)	There was a 18.9% seroprevalence (n = 148) with no association for sex in the tested coyotes. *B. burgdorferi *occurred at higher levels in areas with higher rainfall and in blue oak woodland, montane hardwood, and redwood vegetation regions, and decreased in coastal sagebrush and cropland, providing a finer-scaled analysis within a rural category. *B. burgdorferi *seroprevalence peaked seasonally (June – July and November – April).
Limitations:	While rural coyotes were sampled in this study, urban coyotes do not seem to be included in the study population. When split into subsets, sample sizes are smaller (n = 1–57).

## Appraisal, application and reflection

Lyme disease in the United States is caused by the bacterium *Borrelia burgdorferi *sensu stricto (s.s.) and is vectored by *Ixodes *ticks, namely *Ixodes scapularis *in the eastern United States and *Ixodes pacificus* in the western United States that feed on many animal species including humans (Steere et al., 2016). Throughout the United States, Lyme borreliosis is an important emerging disease causing approximately 30,000 new human cases annually (CDC, 2019). While the majority of cases are in the northeastern/mid-Atlantic United States have occurred in all 50 states (CDC, 2019) and the highly diseased regions are expanding (Steere et al.*,* 2016). This is also driven by climate change, allowing ticks to expand their geographic ranges and increase in abundance in recent decades presenting new and increased health risks to humans and animals (Sonenshine, 2018).

While Lyme disease is more debilitating in humans, dogs and other domestic animals can also contract *Borrelia* infections (Self et al., 2018). In fact, canines can act as sentinels of Lyme disease; testing for exposure to *B. burgdorferi *in dogs can be done during annual wellness visit examinations by veterinarians (Self et al., 2018). Veterinarians can then act as stewards of public health by reporting seroprevalence and cases of Lyme Borreliosis to the local public health department, helping to effectively track the spread of *Ixodes *ticks and *B. burgdorferi* as climate change advances. This serves not only additional animal patients that may present as cases in new areas, but also as a warning flag for human health; public health policy makers can make informed interventions if made aware of new or increasing cases of Lyme disease.

While useful, in private practice domestic animals as a sentinel may be limited because diagnostics is often limited by the permission and finances of pet owners. Coyotes (or other wild canids) are another sentinel species that can be sampled via convenience through animal control agencies, rehab facilities, and roadkill to add to the field’s understanding or Borreliosis risk. While a convenience sample (use of easily available samples or samples on hand) is non-probabilistic and thus does have limitations on the amount it can inform the epidemiologic understanding of a disease, it is a good starting method that limits the challenges of wild species monitoring such as sampling effort and cost of the monitoring efforts. Despite these inherent challenges that exist in monitoring wildlife such as varying population densities and smaller sample sizes, using coyotes as a sentinel is an established practice for other pathogens such as *Anaplasma phagocytophilum *or *Yersinia pestis*, which are both zoonotic vector-borne diseases (Foley et al., 2005; and Brown *et al.,* 2011). Testing coyotes can allow us to characterise disease levels in many different environments because coyotes will utilise and have successfully adapted to not only wilderness and rural but also urban environments (Crooks, 2002). Because coyotes utilise multiple environments and have large home ranges (Gehrt et al., 2009), they could also serve as conduits to take *Borrelia-*infected ticks between habitats or into new areas.

By characterising the exposure and role of *Borrelia *in coyotes, there is potential that daily clinical decisions of veterinarians can be informed. If veterinarians are aware of the prevalence of *Borrelia *in surrounding wildlife and the risk posed to their patients, it can hold an appropriate place on their differential list for clinically affected patients and can be utilised to inform testing and vaccination decisions. At the time of this writing, there is a paucity of information concerning how prevalence varies between urban and rural environments and if this can inform clinical decisions of veterinarians; more research is needed in order to determine if wild coyotes have a higher prevalence of *B. burgdorferi* seroconversion in an urban habitat compared to a rural habitat, and if wild coyotes are indeed a good sentinel species for *B. burgdorferi *in urban environments.

## Methodology

**Search strategy:** 

Databases searched and dates covered:	CAB Abstracts on CAB Direct Platform 1973 – 2021 (included products: CAB ABSTRACTS, VetMed Resource, CABI Full Text, Global Health, Animal Health and Production Compendium (AHPC)) Medline on PubMed 1902 – 2021 (Included products: Medline, in process citations, “ahead of print” citations, out-of-scope citations, journals indexing prior to medline inclusion, pre-1966 citations, PubMed Central, author manuscripts NIH funding, NCBI Bookshelf ) Scopus by Elsevier 1976 – 2021
Search strategy:	CAB Abstracts: Coyote* OR “Canis latrans” Borrelia OR “Lyme Disease” OR “lyme disease” OR “B. burgdorferi” OR “Borrelia spp.” OR “Borrelia sp.” Serology OR seroconversion OR antibodies OR ELISA #1 AND #2 AND #3 PubMed: (((Serology OR seroconversion OR antibodies OR ELISA) AND (Borrelia OR Lyme Disease OR B. burgdorferi OR Borrelia spp. OR Borrelia sp.)) AND (Coyote OR Canis latrans) Scopus: ( TITLE-ABS-KEY ( *serology* OR *seroconversion* OR *antibodies* OR *elisa* OR *"enzyme-linked immunosorbent assay"* ) ) AND ( TITLE-ABS-KEY ( *borrelia* OR *lyme* OR *lyme's* OR *{b. burgdorferi}* ) ) AND ( TITLE-ABS-KEY ( *coyote* OR *coyotes* OR *"canis latrans"* OR *{C. latrans }* ) )
Dates searches performed:	10 Nov 2021

**Exclusion / Inclusion criteria:** 

Exclusion:	Not in English Study area is outside the US *Borrelia burgdorferi* not actually measured / not in coyotes Urban / rural distinction not made for tested coyotes Not peer-reviewed
Inclusion:	English Study area is within the US *Borrelia burgdorferi *measured by authors in coyotes Urban / rural distinction not made for tested coyotes Peer-reviewed

**Search outcome:** 

Database	Number of results	Excluded – Not in English	Excluded – Study area is outside the USA	Excluded – Does not measure Borrelia burgdorferi in coyotes	Excluded – Urban / rural data not included	Excluded – Not peer-reviewed	Total relevant papers
CAB Abstracts	9	0	2	0	6	0	1
PubMed	10	0	2	1	5	0	2
Scopus	10	0	2	1	5	0	2
Total relevant papers when duplicates removed							2

## Conflict of interest

The authors declare no conflicts of interest.

The authors would like to acknowledge Andres Mauricio Lopez-Perez for his mentorship of L. Shultz and proof-reading this manuscript. Additional acknowledgement goes to the 2020 MPM208 Class at University of California, Davis through which this project was undertaken.

## Disclaimer

Knowledge Summaries are a peer-reviewed article type which aims to answer a clinical question based on the best available current evidence. It does not override the responsibility of the practitioner. Informed decisions should be made by considering such factors as individual clinical expertise and judgement along with patient’s circumstances and owners’ values. Knowledge Summaries are a resource to help inform and any opinions expressed within the Knowledge Summaries are the author’s own and do not necessarily reflect the view of the RCVS Knowledge. Authors are responsible for the accuracy of the content. While the Editor and Publisher believe that all content herein are in accord with current recommendations and practice at the time of publication, they accept no legal responsibility for any errors or omissions, and make no warranty, express or implied, with respect to material contained within. For further information please refer to our Terms of Use.
